# Supplementary material for: Unexplained mechanism of subdural hematoma with convulsion suggests nonaccidental head trauma: A multicenter, retrospective study by the Japanese Head injury of Infants and Toddlers study (J-HITs) group
Source: PLoS One. 2022 Nov 3;17(11):e0277103. doi: 10.1371/journal.pone.0277103 (PMC9632799; doi:10.1371/journal.pone.0277103)
Supplement: S1 Appendix — (DOCX) [file pone.0277103.s001.docx]

| **S1 Appendix.**  A list of the 84 subdural hematomas studied in this article with respect to age in months, gender, presence of retinal hemorrhage, presence of other intracranial lesions, the parent's stated injury history, and the final administrative determination of abuse. (DOCX) |  |  |
| --- | --- | --- |

|  | | | |  | |  | | |  | | | |  | |  | | |  |  | |  | | |  | |  | |  |  |
| --- | --- | --- | --- | --- | --- | --- | --- | --- | --- | --- | --- | --- | --- | --- | --- | --- | --- | --- | --- | --- | --- | --- | --- | --- | --- | --- | --- | --- | --- |
| age (months) | | over 6 mos | sex | SDH bilateral or unilateral | | retinal hemorrhage | | brain edema | skull fracture | | contusion | epidural hematoma | subarachnoid hemorrhage | | arachnoid cysts | | epilepsy/seizure | noticification to child guidance center | | | taken into custody | | prosecuted as a criminal case | cause of injury | | categorized as nonaccidental trauma in this study | |  | |
| 4 | | no | male | bilateral | | yes | | yes | yes | | no | no | no | | no | | yes | yes | | | no | | unknown | Other falls from <2m | | no | |  | |
| 4 | | no | male | bilateral | | yes | | no | no | | no | no | yes | | no | | yes | yes | | | no | | no | Other falls from <2m | | no | |  | |
| 9 | | yes | male | unilateral | | yes | | no | no | | no | no | no | | no | | yes | yes | | | no | | no | Other falls from <2m | | no | |  | |
| 0 | | no | female | bilateral | | yes | | yes | no | | no | no | no | | no | | no | yes | | | no | | no | Other falls from <2m | | no | |  | |
| 1 | | no | male | unilateral | | yes | | no | yes | | no | no | yes | | no | | no | yes | | | yes | | yes | Unexplained coma | | yes | |  | |
| 2 | | no | male | unilateral | | yes | | no | no | | no | no | no | | no | | no | yes | | | yes | | yes | Unexplained coma | | yes | |  | |
| 8 | | yes | male | bilateral | | yes | | no | no | | no | no | no | | no | | no | yes | | | yes | | yes | Unexplained coma | | yes | |  | |
| 34 | | yes | male | unilateral | | yes | | yes | no | | no | no | no | | no | | no | yes | | | yes | | unknown | Unexplained coma | | yes | |  | |
| 1 | | no | male | bilateral | | yes | | yes | no | | no | no | no | | no | | no | yes | | | yes | | no | Unexplained coma | | yes | |  | |
| 8 | | yes | male | unilateral | | yes | | no | no | | no | no | no | | no | | no | yes | | | yes | | no | Unexplained coma | | yes | |  | |
| 3 | | no | male | bilateral | | yes | | no | no | | no | no | no | | no | | no | yes | | | no | | no | Unexplained coma | | no | |  | |
| 3 | | no | female | bilateral | | yes | | no | no | | no | no | yes | | no | | yes | yes | | | yes | | no | Parents dropped | | yes | |  | |
| 4 | | no | male | bilateral | | yes | | no | yes | | no | no | yes | | no | | no | yes | | | yes | | unknown | Falling while being held by a parent | | yes | |  | |
| 4 | | no | male | bilateral | | yes | | no | yes | | no | no | no | | no | | yes | yes | | | yes | | no | Falling while being held by a parent | | yes | |  | |
| 7 | | yes | female | unilateral | | yes | | no | no | | no | no | no | | no | | yes | yes | | | yes | | no | Falling while being held by a parent | | yes | |  | |
| 5 | | no | female | unilateral | | yes | | no | no | | no | no | no | | no | | no | yes | | | yes | | no | Falling while being held by a parent | | yes | |  | |
| 2 | | no | female | bilateral | | yes | | no | yes | | no | no | no | | no | | no | yes | | | no | | no | Falling while being held by a parent | | no | |  | |
| 4 | | no | male | unilateral | | yes | | no | yes | | no | no | yes | | no | | yes | yes | | | yes | | yes | Confessed abuse | | yes | |  | |
| 3 | | no | male | bilateral | | yes | | no | no | | yes | no | no | | no | | yes | yes | | | yes | | yes | Confessed abuse | | yes | |  | |
| 5 | | no | female | bilateral | | no | | no | no | | yes | no | no | | no | | yes | yes | | | yes | | yes | Confessed abuse | | yes | |  | |
| 4 | | no | female | bilateral | | yes | | yes | no | | no | no | no | | no | | yes | yes | | | yes | | yes | Confessed abuse | | yes | |  | |
| 4 | | no | male | bilateral | | yes | | no | no | | no | no | no | | no | | yes | yes | | | yes | | yes | Confessed abuse | | yes | |  | |
| 20 | | yes | male | unilateral | | yes | | yes | no | | no | no | no | | no | | yes | yes | | | no | | yes | Confessed abuse | | yes | |  | |
| 3 | | no | male | bilateral | | no | | no | no | | no | no | no | | no | | yes | yes | | | yes | | unknown | Confessed abuse | | yes | |  | |
| 3 | | no | male | unilateral | | no | | no | yes | | no | no | no | | no | | no | yes | | | yes | | unknown | Confessed abuse | | yes | |  | |
| 4 | | no | male | unilateral | | no | | yes | no | | no | no | no | | no | | no | yes | | | yes | | unknown | Confessed abuse | | yes | |  | |
| 30 | | yes | female | unilateral | | yes | | yes | no | | no | no | no | | no | | no | yes | | | no | | unknown | Confessed abuse | | yes | |  | |
| 8 | | yes | female | unilateral | | yes | | yes | no | | no | no | no | | no | | yes | yes | | | yes | | no | Confessed abuse | | yes | |  | |
| 2 | | no | male | unilateral | | yes | | yes | no | | no | no | no | | no | | yes | yes | | | no | | no | Confessed abuse | | yes | |  | |
| 15 | | yes | male | unilateral | | no | | no | no | | no | no | no | | no | | yes | yes | | | no | | no | other head injury | | no | |  | |
| 15 | | yes | male | unilateral | | yes | | no | no | | no | no | no | | no | | no | yes | | | no | | no | other head injury | | no | |  | |
| 2 | | no | female | bilateral | | yes | | yes | no | | no | no | yes | | no | | yes | yes | | | yes | | yes | Unexplained convulsions | | yes | |  | |
| 4 | | no | male | bilateral | | yes | | no | no | | yes | no | no | | no | | yes | yes | | | yes | | yes | Unexplained convulsions | | yes | |  | |
| 2 | | no | male | bilateral | | no | | yes | yes | | no | no | no | | no | | yes | yes | | | yes | | yes | Unexplained convulsions | | yes | |  | |
| 2 | | no | female | bilateral | | yes | | no | yes | | no | no | no | | no | | yes | yes | | | yes | | yes | Unexplained convulsions | | yes | |  | |
| 6 | | yes | female | bilateral | | yes | | no | no | | no | no | no | | no | | yes | yes | | | yes | | yes | Unexplained convulsions | | yes | |  | |
| 1 | | no | female | unilateral | | yes | | no | yes | | no | no | yes | | no | | yes | yes | | | yes | | unknown | Unexplained convulsions | | yes | |  | |
| 3 | | no | male | bilateral | | yes | | no | no | | no | no | yes | | no | | yes | yes | | | yes | | unknown | Unexplained convulsions | | yes | |  | |
| 1 | | no | male | bilateral | | no | | no | no | | yes | yes | no | | no | | yes | yes | | | yes | | unknown | Unexplained convulsions | | yes | |  | |
| 1 | | no | male | unilateral | | yes | | yes | yes | | yes | no | no | | no | | yes | yes | | | yes | | unknown | Unexplained convulsions | | yes | |  | |
| 2 | | no | male | unilateral | | yes | | yes | yes | | no | no | no | | no | | yes | yes | | | yes | | unknown | Unexplained convulsions | | yes | |  | |
| 7 | | yes | female | unilateral | | yes | | no | no | | no | no | no | | no | | yes | yes | | | yes | | unknown | Unexplained convulsions | | yes | |  | |
| 4 | | no | male | unilateral | | yes | | no | no | | no | no | no | | no | | yes | yes | | | yes | | unknown | Unexplained convulsions | | yes | |  | |
| 2 | | no | female | unilateral | | no | | no | no | | no | no | yes | | no | | yes | yes | | | yes | | no | Unexplained convulsions | | yes | |  | |
| 2 | | no | female | bilateral | | yes | | no | no | | no | no | no | | no | | yes | yes | | | yes | | no | Unexplained convulsions | | yes | |  | |
| 4 | | no | female | bilateral | | yes | | no | no | | no | no | no | | no | | yes | yes | | | yes | | no | Unexplained convulsions | | yes | |  | |
| 2 | | no | male | bilateral | | yes | | no | no | | no | no | no | | no | | yes | yes | | | yes | | no | Unexplained convulsions | | yes | |  | |
| 6 | | yes | male | bilateral | | yes | | no | no | | no | no | no | | no | | yes | yes | | | yes | | no | Unexplained convulsions | | yes | |  | |
| 6 | | yes | female | unilateral | | no | | no | no | | no | no | no | | no | | yes | yes | | | yes | | no | Unexplained convulsions | | yes | |  | |
| 7 | | yes | female | bilateral | | yes | | no | no | | no | no | no | | no | | yes | yes | | | no | | no | Unexplained convulsions | | no | |  | |
| 8 | | yes | male | unilateral | | yes | | yes | no | | no | no | no | | no | | no | yes | | | no | | no | Falls from >2m | | no | |  | |
| 4 | | no | male | unilateral | | yes | | yes | yes | | no | no | no | | no | | yes | no | | | no | | no | Motor vehicle accidents | | no | |  | |
| 4 | | no | male | unilateral | | yes | | no | no | | no | no | no | | no | | yes | no | | | no | | no | Motor vehicle accidents | | no | |  | |
| 2 | | no | female | unilateral | | no | | no | no | | no | no | no | | no | | no | no | | | no | | no | Motor vehicle accidents | | no | |  | |
| 41 | | yes | male | unilateral | | yes | | yes | yes | | no | no | no | | no | | no | yes | | | no | | no | Falls from >2m | | no | |  | |
| 15 | | yes | male | unilateral | | yes | | yes | no | | no | no | no | | no | | yes | yes | | | yes | | unknown | Self-inflicted fall | | yes | |  | |
| 9 | | yes | male | unilateral | | yes | | no | no | | no | no | no | | no | | yes | yes | | | yes | | no | Self-inflicted fall | | yes | |  | |
| 9 | | yes | male | unilateral | | yes | | no | no | | no | no | no | | no | | yes | yes | | | yes | | no | Self-inflicted fall | | yes | |  | |
| 9 | | yes | male | unilateral | | yes | | no | no | | no | no | no | | no | | no | yes | | | yes | | no | Self-inflicted fall | | yes | |  | |
| 9 | | yes | male | unilateral | | yes | | no | no | | no | no | no | | no | | no | yes | | | yes | | no | Self-inflicted fall | | yes | |  | |
| 8 | | yes | male | unilateral | | yes | | yes | no | | no | no | no | | no | | yes | yes | | | no | | no | Self-inflicted fall | | no | |  | |
| 9 | | yes | female | unilateral | | yes | | no | no | | no | no | no | | no | | yes | yes | | | no | | no | Self-inflicted fall | | no | |  | |
| 8 | | yes | male | unilateral | | yes | | no | no | | no | no | no | | no | | yes | yes | | | no | | no | Self-inflicted fall | | no | |  | |
| 8 | | yes | male | unilateral | | yes | | no | no | | no | no | no | | no | | yes | yes | | | no | | no | Self-inflicted fall | | no | |  | |
| 10 | | yes | male | unilateral | | yes | | no | no | | no | no | no | | no | | yes | yes | | | no | | no | Self-inflicted fall | | no | |  | |
| 10 | | yes | male | unilateral | | yes | | no | no | | no | no | no | | no | | yes | yes | | | no | | no | Self-inflicted fall | | no | |  | |
| 12 | | yes | female | unilateral | | yes | | no | yes | | no | no | no | | no | | no | yes | | | no | | no | Self-inflicted fall | | no | |  | |
| 10 | | yes | male | unilateral | | yes | | yes | no | | no | no | no | | no | | no | yes | | | no | | no | Self-inflicted fall | | no | |  | |
| 7 | | yes | male | unilateral | | yes | | no | no | | no | no | no | | no | | no | yes | | | no | | no | Self-inflicted fall | | no | |  | |
| 10 | | yes | male | unilateral | | yes | | no | no | | no | no | no | | no | | no | yes | | | no | | no | Self-inflicted fall | | no | |  | |
| 17 | | yes | male | unilateral | | no | | no | no | | no | no | no | | yes | | no | no | | | no | | no | Bicycle accidents | | no | |  | |
| 10 | | yes | male | unilateral | | yes | | yes | no | | no | no | no | | no | | no | yes | | | yes | | no | Falling from a bed or sofa | | yes | |  | |
| 20 | | yes | male | unilateral | | yes | | yes | no | | no | no | no | | no | | yes | yes | | | no | | no | Falling from a bed or sofa | | no | |  | |
| 12 | | yes | male | unilateral | | yes | | no | no | | no | no | no | | no | | yes | yes | | | no | | no | Falling from a bed or sofa | | no | |  | |
| 8 | | yes | male | unilateral | | yes | | yes | no | | no | no | no | | no | | no | yes | | | no | | no | Falling from a bed or sofa | | no | |  | |
| 9 | | yes | male | unilateral | | no | | no | no | | no | no | no | | no | | no | yes | | | no | | no | Falling from a bed or sofa | | no | |  | |
| 2 | | no | male | bilateral | | yes | | no | yes | | no | no | yes | | no | | no | yes | | | yes | | unknown | Other head injury | | yes | |  | |
| 5 | | no | male | bilateral | | yes | | no | no | | no | no | no | | no | | yes | yes | | | yes | | no | Other head injury | | yes | |  | |
| 8 | | yes | male | unilateral | | yes | | no | no | | no | no | no | | no | | no | yes | | | no | | no | Other head injury | | no | |  | |
| 4 | | no | male | bilateral | | yes | | no | yes | | yes | no | no | | no | | no | yes | | | yes | | no | other unexplained event | | yes | |  | |
| 3 | | no | male | bilateral | | yes | | no | no | | no | no | no | | no | | no | yes | | | yes | | no | other unexplained event | | yes | |  | |
| 0 | | no | female | unilateral | | yes | | yes | no | | no | no | no | | no | | no | no | | | no | | no | Birth injury | | no | |  | |
| 0 | | no | male | unilateral | | no | | yes | no | | no | no | no | | no | | no | no | | | no | | no | Birth injury | | no | |  | |
| 0 | | no | male | unilateral | | no | | no | no | | no | no | no | | no | | no | no | | | no | | no | Birth injury | | no | |  | |
