# Supplementary material for: Unexplained mechanism of subdural hematoma with convulsion suggests nonaccidental head trauma: A multicenter, retrospective study by the Japanese Head injury of Infants and Toddlers study (J-HITs) group
Source: PLoS One. 2022 Nov 3;17(11):e0277103. doi: 10.1371/journal.pone.0277103 (PMC9632799; doi:10.1371/journal.pone.0277103)
Supplement: Attachment — Submitted filename: Response to Reviewers.docx [file pone.0277103.s003.docx]

Response to Reviewers

**Reviewer #1:**

**Comment #1** p5. add space between the and disease at the end of line 2.

**Author’s Response:** The text has been revised according to the reviewer`s suggestion.

**Comment #2** Add a period at the end of the paragraph "and a statement regarding patient consent."

**Author’s Response:** The text has been revised according to the reviewer`s suggestion.

**Comment #3** p6. In table 2, please align the cause of injury to the left to aid readability.

**Author’s Response:** The table has been revised according to the reviewer`s suggestion.

**Comment #4** p7. In table 3, please increase spacing in the heading so letters are not dropped to the next line.

**Author’s Response:** The table has been revised according to the reviewer`s suggestion.

**Comment #5** p8. Please align all paragraphs to the left.

**Author’s Response:** The paragraphs has been revised according to the reviewer`s suggestion.

**Comment #6** p9. Please add a space in the second paragraph of the discussion between accidents and (10).

**Author’s Response:** The paragraphs has been revised according to the reviewer`s suggestion.

**Comment #7** p10.Change second line to say "...inconsistent with the described injury mechanism, there is consensus that the medical validity..."

**Author’s Response:** The text has been revised according to the reviewer`s suggestion.

**Comment #8** p11. remove space at end of sentence in line 3.

**Author’s Response:** The text has been revised according to the reviewer`s suggestion.

**Comment #9** Correct spelling to "were" in the line 3 of the third paragraph.

**Author’s Response:** The text has been revised according to the reviewer`s suggestion.
